# Shear characteristics and shear strength model of rock mass structural planes

**DOI:** 10.1038/s41598-022-17998-z

**Published:** 2022-08-10

**Authors:** Shan Dong, Yulin Peng, Zhichun Lu, Heng Zhang, Weihua Hou, Aijun Su

**Affiliations:** 1 Badong National Observation and Research Station of Geohazards, China University of Geosciences, Wuhan, 430074 China; 2 Three Gorges Research Center for Geohazards, China University of Geosciences, Wuhan, 430074 China

**Keywords:** Civil engineering, Solid Earth sciences

## Abstract

Accurately determining the shear strength of structural planes is crucial for evaluating the stability of rock masses. The shear test using the sawtooth structural plane usually captures the main influencing factors of its shear characteristics. In this study, the two-dimensional particle flow code (PFC2D) numerical simulation method was used to conduct shear tests on the sawtooth structural planes of rock masses with undulant angles of 10°, 20°, and 30°, respectively. With the increase in normal stress and the undulant angle, the shear failure of the structural planes was found to no longer be pure slip failure or shear failure but accompanied by a compression-induced fracture phenomenon. Based on the analysis of the shear test results, a peak shear strength model considering different undulant angles and normal stresses was proposed, and the hyperbolic function post-peak shear strength model was improved. The peak shear strength obtained from the physical direct shear tests was compared with those calculated using the proposed model, Parton model, and Shen model. The calculation error under low and high normal stress of the proposed method was found to be within an acceptable range. Additionally, when calculating the peak shear strength of a structural plane under high normal stress, applying the calculation method proposed in this study is a better option than applying the other models. Furthermore, although the variation trend of the post-peak shear strength was similar to that of the experimental results, the values obtained using the hyperbolic variation model were too large. The variation trend of the post-peak shear strength obtained using the improved function was essentially consistent with the experimental results, and the calculated values were close to the experimental results. The systematic research on the shear strength calculation model of rock mass structural planes contributes to the theoretical research of rock mass mechanics, and this study can act as a guide for landslide prediction and control projects.

## Introduction

Rough and undulating rock mass structural planes widely develop along rocky slopes. Structural planes reduce the integrity of a rock mass and improve the physical and mechanical properties of the rock mass anisotropy, discontinuity, and heterogeneity, which control the mechanical properties of the rock mass^1–7^. Furthermore, the shear mechanical properties of these structural planes substantially influence the stability of these slopes^8–10^. For example, the Jiweishan and Qianjiangping landslides were caused by the further weakening of the mechanical properties along the weak interlayer structural planes at the bottom of a sliding body that eventually developed into a sliding surface^11–18^. Therefore, the shear strength of rock mass structural planes is one of the most important indices in the evaluation of the stability of rock masses. Additionally, the shear characteristics and a shear strength model of rock mass structural planes are crucial for theoretical research and engineering practices.

The shear characteristics and empirical models of the shear strength along rock mass structural planes have attracted extensive attention, and ample research has been conducted on the topic. Patton^19^ used a direct shear test and discovered that the peak shear strength of a structural plane is related to the normal stress, and proposed the popular Patton linear formula. However, the peak shear strength envelopes for non-planar rock joints are nonlinear. There are some differences between the Patton linear relationship and the actual rough joint surface shearing situation. Ladanyi and Archambault^20^ established a peak shear strength model for rock mass structural planes containing a rock bridge by combining the effects of friction, dilatancy, cohesion, and rock bridge strength considering the shear sliding mechanism of natural rock mass structural planes. But the method proposed by Ladanyi and Archambault^20^ is more accurate when only the undulant angle is considered. Based on the study by Ladanyi and Archambault^20^ and using numerical simulations, Huang et al.^21^ established an empirical shear strength formula that considers the slip and shear effects of serrated rock mass structural planes. By analysing the relationships between structural plane roughness, normal stress, and the dilatancy angle, the Barton-Bandis (B-B) shear strength model was proposed^22,23^. Based on the Barton-Bandis (B-B) shear strength model, many improved models for shear strength estimation of structural planes with parameters including roughness and fluctuation characteristics have been put forward in recent years^24–26^. Shen and Zhang^27^ amended the shear strength model using dilatancy and shearing effects by introducing a correction factor for the internal friction angle and the comprehensive cohesion of rock mass structural planes. Ueng et al.^28^ and Vallier et al.^29^ further verified the substantial change in the ratio of the shear strength to the normal stress of the structural planes owing to an increase in the normal stress. Although substantial research has been conducted on the shear characteristics of structural surfaces, which has contributed to defining the shear mechanical properties and parameter values of rock mass structure planes, studies on the most essential mechanical index, namely the shear strength of structural surfaces, are limited.

The interference from manual excavation and seismic load may not cause a rock mass to suddenly destabilise owing to displacement and deformation, but instead, the rock mas may gradually stabilise. Additionally, the post-peak shear strength of the structural plane controls the stability of the rock mass^30–34^. Additionally, several studies have been conducted on the post-peak shear strength of rock mass structural planes. For instance, Saeb and Amadei^35,36^ established a shear stress–strain model for the shear process by graphically and analytically investigating the shear stress–strain curve of a structural plane under normal stress. These authors were the first to propose a linear attenuation model of the post-peak shear strength. Simon^37^ used a simple exponential function model to describe the entire shear stress–displacement process and proposed a complete stress–displacement surface model to describe the nonlinear shear stress–displacement relationship. Lee et al.^38^ reported that the decrease in the experimental curve of the shear stress from the peak to the residual shear was similar to a hyperbolic variation. Grasselli and Egger^39^ adopted a hyperbolic variation to propose a model for post-peak shear strength. Indraratna et al.^40^ introduced the dilation rate ($\dot{v}$) into the shear stress–shear displacement model of the structural plane, thereby obtaining a dynamic shear stress–displacement model of the plane under constant normal stiffness conditions. However, the existing post-peak shear strength calculation model cannot accurately reflect the nonlinear variation trend of post-peak shear stress–shear displacement. Therefore, the calculation accuracy of this model needs to be improved.

Previous studies have mainly examined the shear strength of structural planes based on experimental and theoretical estimations. In experimental estimations, the shear strength law curve and related mechanical mechanism were investigated based on numerous direct shear tests on structural planes. Finally, the test results were fitted to the shear strength test estimation formula. In contrast, in theoretical estimations, the shear strength along the structural plane was theoretically analysed, after which a theoretical model was proposed. Finally, the theoretical model was verified and revised using related tests. Experimental methods mainly included physical direct shear tests and numerical simulations. The physical direct shear test can only obtain the mechanical properties of the structural plane through macro analysis, and observing the micro-failure phenomenon along the structural plane during the shear process is difficult. However, the numerical calculation method can overcome many difficulties related to the physical direct shear test, and the meso-failure characteristics of the shear process can be directly observed^41–45^. The particle flow code (PFC) method proposed by Cundall^46^ can simulate the adhesion and friction between rock mineral particles in a mesoscale, thereby avoiding the reliance on empirical parameters to obtain the macroscopic composition of the model. At present, PFC is applied widely for simulating the mechanical properties of rocks^47–52^.

In this study, based on the two-dimensional PFC (PFC2D) calculation program, a numerical direct shear test was performed on rock mass structural planes by considering different undulant angles and normal stresses. The failure characteristics of the structural plane were analysed. Further, the evolution characteristics of the shear stress with the change in the shear displacement were analysed in-depth to improve the peak and post-peak shear strength models for rock mass structural planes. The systematic research on the shear strength calculation model of rock mass structural planes enriches the basic theoretical research of rock mass mechanics. Simultaneously, this study can act as a guide for landslide prediction and control projects.

## Parameter calibration

Because the meso-level parameters involved in PFC2D possess internal randomness and a complicated relationship with macro-mechanical properties, the calibration of these parameters is crucial for ensuring the accuracy of the test. To ensure the highest level of consistency between the numerical test results with the results of the physical test, the parameters should be calibrated using the macroscopic physical test results before conducting the numerically simulated direct shear test on the structural plane. In the macroscopic physical test, the trial-and-error method is used to repeatedly modify the meso-parameters until the results of the numerical simulation and the test results are within the error range. When the macro-mechanical properties are consistent with the physical test results, the calibration parameters are considered to be optimised^53–56^.

### Physical shear test

**Figure 1:**
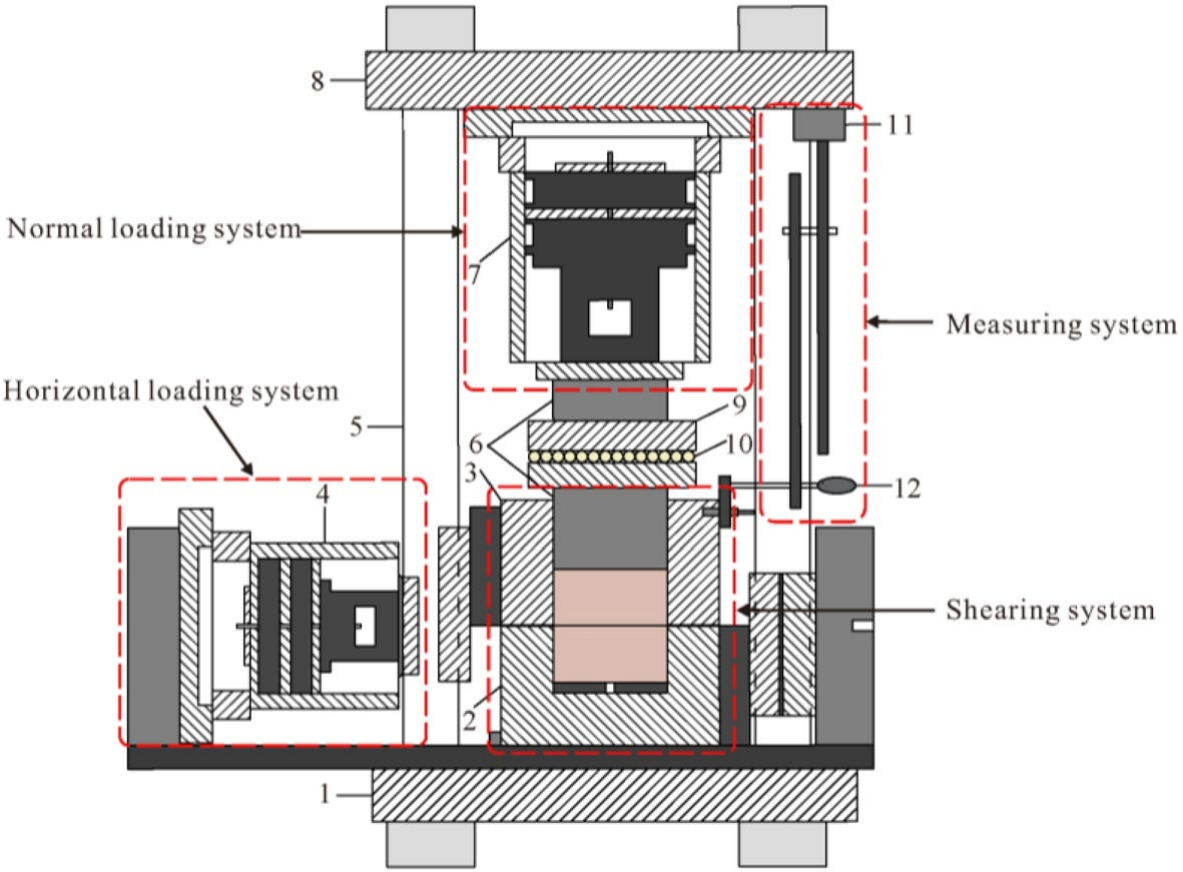
Portable rock mechanical property multifunctional test device^57^. 1: bottom frame baffle; 2: lower shear box; 3: upper shear box; 4: horizontal jack loading; 5: vertical frame baffle; 6: force transmission device; 7: vertical jack loading; 8: top frame baffle; 9: skateboards; 10: ball bearings; 11: magnetic stand; 12: dial gauge.

An intact Jurassic red-bed sandstone rock sample (with dimensions of 100 mm × 100 mm × 100 mm) and a red-bed sandstone rock sample with a flat structural plane (dimensions of the upper and lower parts were 100 mm × 100 mm × 50 mm each) were used for the physical direct shear tests. The portable rock mechanical performance multifunctional test device that was independently developed by the Chengdu University of Technology, was used to perform the physical direct shear tests. Additionally, this test device mainly comprises normal loading, horizontal loading, shearing, and measuring systems (Fig. 1). The shear tests were conducted under the normal stress values of 1, 2, and 3 MPa. During the shearing process, the normal load remained unchanged and the shear load was applied step by step. Simultaneously, the shear and normal displacement under each level of shear load were measured and recorded. Figures 3 and 4 show the shear stress–shear displacement curves obtained using the physical direct shear tests performed on the intact sandstone rock sample and the rock sample with a flat structural plane.

### Parameter calibration

**Figure 2:**
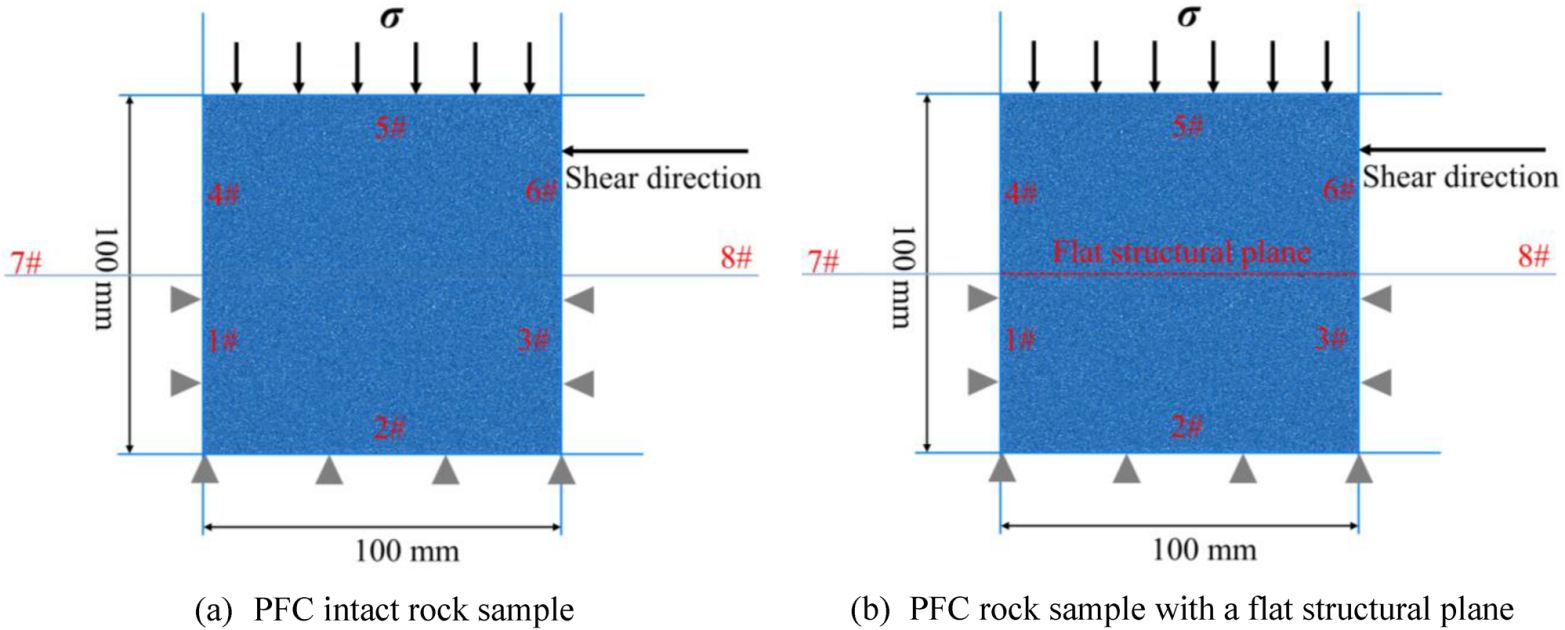
PFC intact rock sample and the PFC rock sample with a flat structural plane.

**Figure 3:**
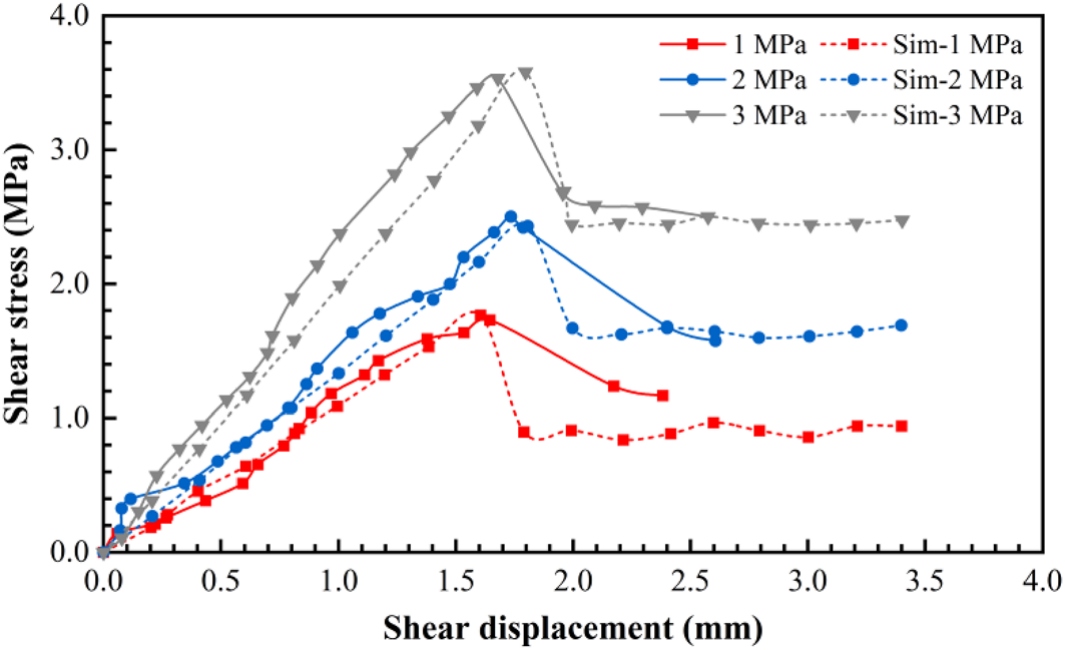
Comparison of the shear stress–displacement curves obtained from the physical direct shear tests and simulations on the sandstone sample without a structural plane.

To construct the PFC rock sample, uniformly distributed particles with diameters ranging from 0.15 to 0.3 mm were generated in the wall box, the size of which was identical to that of the actual sample (100 mm × 100 mm), by setting an initial porosity of 0.15. Subsequently, the particles were re-balanced under an isotropic compressive stress of 100 kPa, and the PFC built-in contact model of the linear parallel bond model was used to bond the particles in contact. A parallel bond imparts the mechanical behaviour of a finite-sized piece of cement-like material deposited between the two contacting particles, and the granular material becomes rock-like after bonding^53^. Additionally, a joint was added to the bonded assembly by applying the PFC built-in contact model of the smooth-joint model to selected contacts. The smooth-joint model was used to simulate the mechanical behaviour of a rock joint^53^. The PFC intact rock sample and the PFC rock sample with a flat joint are shown in Figs. 2 and 3.

The shear rate (${V}_{s}$) in the simulation was proportional to the length of the specimen and was calculated using the following equation:

1 $${V}_{s}=\frac{5000 \times 0.5 \% \times L}{60}$$

where L is the sample length. The calculated ${V}_{s}$ was approximately 0.7 mm/min.

**Figure 4:**
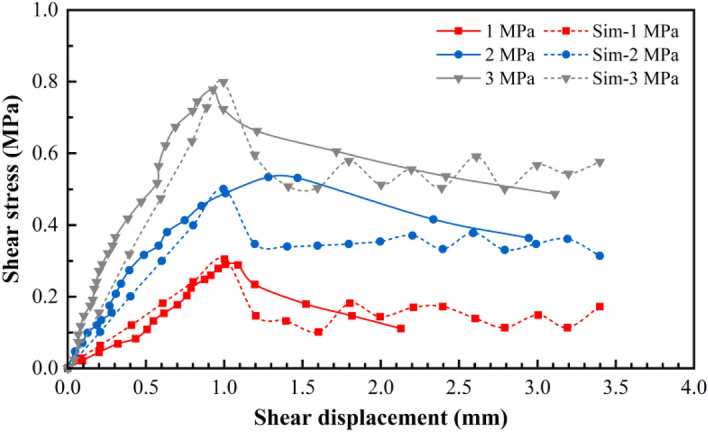
Shear stress–displacement curves obtained from the simulations of the flat structural plane in the sandstone sample.

During the shearing process, the data, including shear stress and normal displacement, were recorded for every 0.5% shear strain, and the test was terminated when the shear displacement was 3.5 mm with reference to the physical direct shear test results. Using trial and error, the PFC parameters were adjusted repeatedly until the shear stress–displacement curve obtained from the simulation fitted well with that obtained from the physical direct shear test (Figs. 3 and 4). Tables 1, 2 and 3 list the calibration parameters.

**Table 1:** Microscopic parameters of the test particles used in the numerical simulations.

Lithology	Minimum particle radius (mm)	Maximum particle radius (mm)	Particle density (g/cm^3^)	Particle contact modulus (GPa)	Normal and tangential stiffness ratio of particles	Particle friction coefficient
Sandstone	0.15	0.3	2.65	1.0	2.0	0.5

**Table 2:** Meso-parameters of the parallel bonding model in the numerical simulations.

Linear or parallel connection elastic modulus (GPa)	Linear or parallel bond stiffness ratio	Bonding distance (mm)	Contact friction coefficient	Average contact tensile strength (MPa)	Standard deviation of contact tensile strength (MPa)	Standard deviation of contact cohesion (MPa)	Average contact cohesion (MPa)
1.0	2.0	0.5	0.5	10	5	5	10

**Table 3:** Meso-parameters of the smooth-joint model in the numerical simulations.

Normal stiffness (GPa)	Normal and tangential stiffness ratio	Contact friction coefficient	Dilatancy angle (°)	Average contact tensile strength (Pa)	Standard deviation of contact tensile strength (Pa)	Average contact cohesion (Pa)	Standard deviation of contact cohesion (Pa)
1.0	2.0	0.5	0.0	0.0	0.0	0.0	0.0

## Shear test on the structural plane

The morphological characteristics of the structural plane mainly affect its shear strength. The surface morphology of a regularly undulating structural plane can generally be divided into three types according to geometric characteristics: straight, sawtooth, and stepped. The shear strength of a real unfilled structural plane is mainly composed of the following three aspects: (1) the frictional force provided by the basic friction angle, (2) ascent angle owing to the surface morphology, and (3) gnawing force owing to the abrasion or shearing of the surface protrusions. When the sawtooth structural plane is sheared, distinct mechanical effects, such as the ascent and gnawing effects may occur. This can quantitatively describe the effect of the undulant angle on the mechanical properties of the structural plane. The existing research results^48,54–56^ demonstrate that although differences exist between the sawtooth and straight structural planes, the shear test using the sawtooth structural plane usually captures the main influencing factors of its shear characteristics, such as roughness and undulation, with certain rationality. Therefore, the regular sawtooth structural plane samples were selected for the structural plane simulations to examine the macro and meso-failure processes, as well as the shear stress evolution characteristics of the rock mass structural plane. In this simulation, the undulant angles of the regular sawtooth structural plane were 10°, 20°, and 30°.

**Figure 5:**
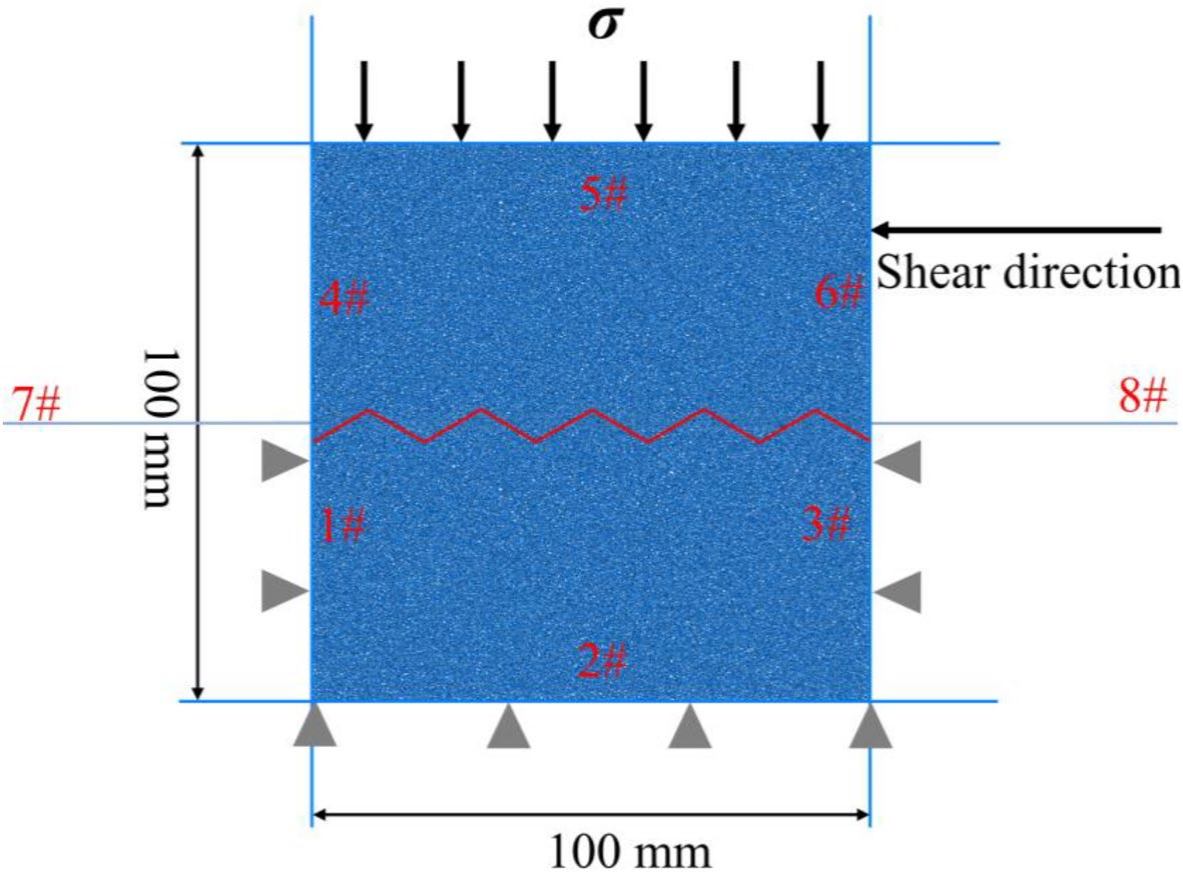
Diagram of the particle flow code (PFC) numerical simulation model.

According to the direct shear model of the upper and lower shear boxes on a 2D plane established by Fu^58^ and Zhang et al.^59^, a total of eight walls were established to simulate the shear box. Among them, walls #1–3 formed the lower shear box, whereas walls #4–6 formed the upper shear box, as shown in Fig. 5. The height and width of the upper and lower shear boxes were 50 and 100 mm, respectively (Fig. 5). Additionally, walls #7 and 8 acted as wing walls on both sides to prevent the overflow of the particles (Fig. 5). A total of 50,964 particles were generated randomly in this model with their radii evenly distributed in the range of 0.15–0.3 mm, a density of 2650 kg/m^3^, and porosity of 0.16. A parallel contact model was used as the contact constitutive model between particles, and a smooth-joint contact model was used to simulate the rock mass structural plane.

In this test, the normal stress was measured at 0.1, 0.5, 1, 2, and 3 MPa. Wall #5 was controlled using servo control with constant normal stress being applied to the sample. Additionally, a displacement control method was used to apply a shear load. A series of structural plane shear tests conducted by Barton and Choubey^60^ revealed that the peak shear strength of a structural plane causes the shear displacement to be approximately 1% of the length of the structural surface. However, when the shear strength decreases to the residual strength, the shear displacement is approximately 10% of the length of the structural plane^60^. Therefore, the shear target displacement was set to 10% of the length of the structural plane, which was 10 mm. The wall below the shear plane was fixed; however, that above the shear plane moved periodically at a uniform speed of 0.7 mm/min. During the shear process, the data, including the normal stress, horizontal displacement, and shear stress, were recorded at every horizontal shear displacement of 0.4%. Additionally, images were recorded at the corresponding times.

## Analysis of the numerical simulation results

### Analysis of the shear failure characteristics

According to the physical direct shear test results, when the undulant angle of the structural plane and normal stress were small, the failure model of the structural plane mainly included slip failure. In contrast, with the increase in the undulant angle of the structural plane and normal stress, the failure model of the structural plane mainly included shear failure. However, when the values of undulant angle and normal stress were large, shear failure accompanied by a compression-induced fracture phenomenon gradually occurred. This phenomenon has been reported in many previous studies^61–68^. For example, as the structural plane with the undulant angle of 20° when the normal stress is 3 MPa, and the structural plane undulant angle of 30° when the normal stress is 2 and 3 MPa. The failure mode of the shear failure and the compression-induced fracture phenomenon was accompanied by tension and crushing, which was considerably different from the slip failure and pure shear failure modes.

**Figure 6:**
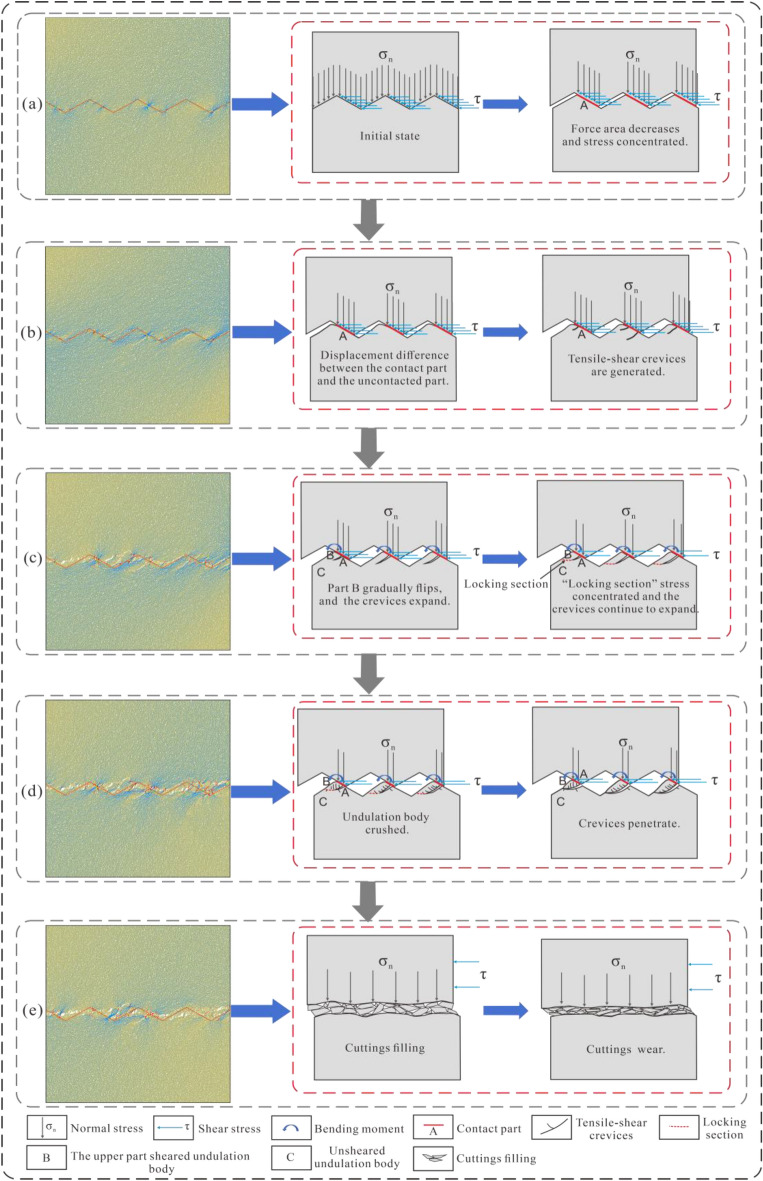
Process diagram of shear failure accompanied by the compression-induced fracture mechanism. The deformation and failure mechanism of the undulating body on the upper part of the rock mass sample is essentially identical to that on the lower part of the rock mass sample.

According to the numerical simulations, the shear failure accompanied by the compression-induced fracture phenomenon was as follows:

1. In the initial stages, as the shear displacement increased, the force area of the structural plane decreased with the stress concentrated at the contact (Fig. 6a).
2. A difference was observed in the displacement between the contacted and uncontacted parts, thereby resulting in tensile stress at the contact and tensile-shear crevices being generated (Fig. 6b).
3. After the appearance of tensile-shear crevices, part B in Fig. 6c tended to flip owing to the bending moment. Simultaneously, as the shear stress increased, the cracks continued to expand and gradually became parallel to the direction of the maximum principal stress (Fig. 6c).
4. As the shear displacement continued to increase, part B gradually became perpendicular to the maximum principal stress, thereby crushing the undulating body. Simultaneously, affected by the stress concentration at the ''locking section'' and crushing zone, the crevices penetrated the undulating body, thereby resulting in shearing and damage (Fig. 6d).
5. After the structural surface was damaged, the shear strength of the structural plane in the subsequent shearing process was mainly derived from friction on the contact surface of the structural plane, structural plane and cutting fill, and friction between the cutting fills. The volume and distribution of the cutting fill greatly influenced the shear strength (Fig. 6e).

### Evolution characteristics of shear stress

**Figure 7:**
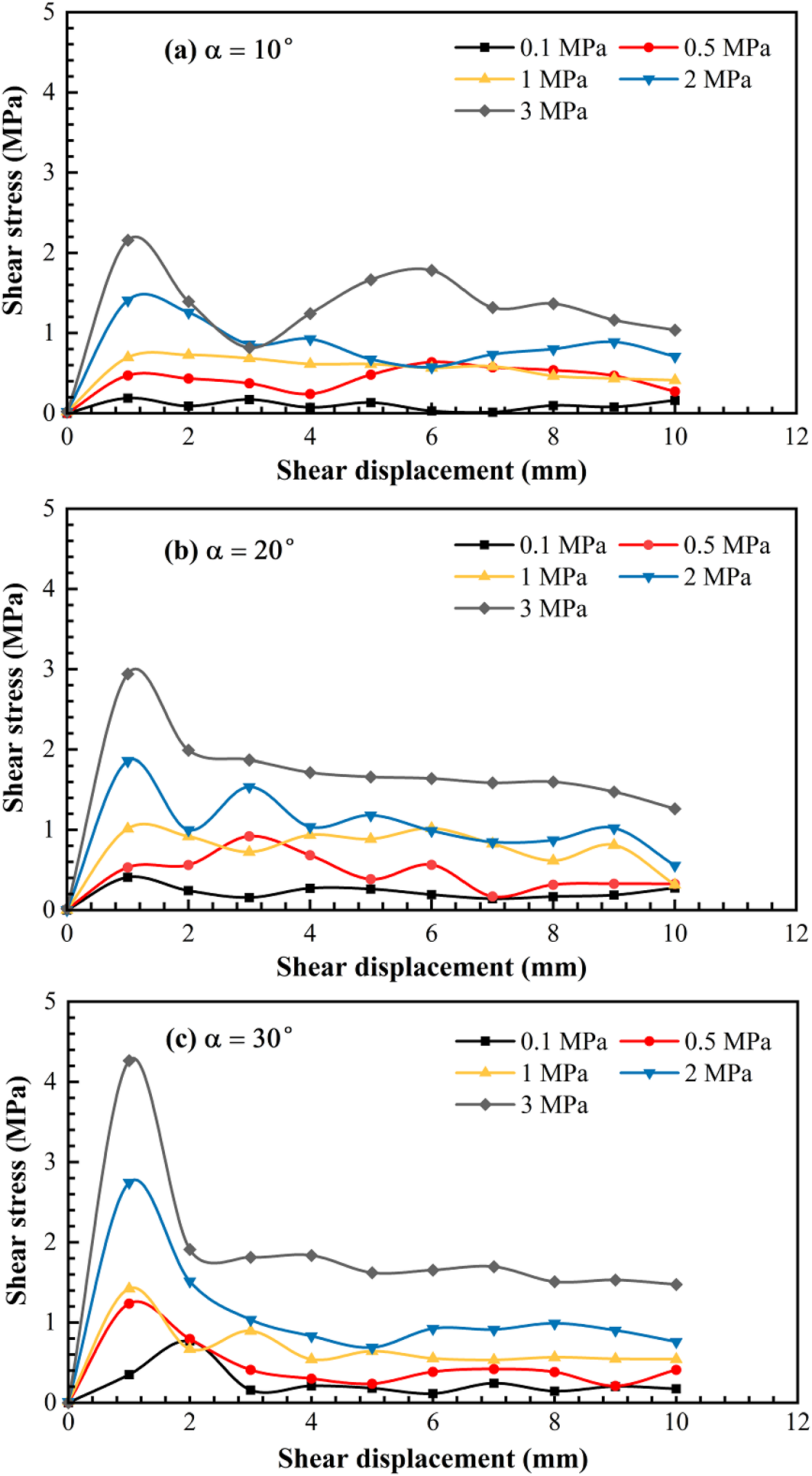
Shear stress–displacement curves of three structural planes under different normal stresses ($\mathrm{\alpha }$ is the undulant angle of the structural plane).

The shear stress-displacement curves are shown in Fig. 7. When the undulant angle was 10°, the normal stress was 0.1, 0.5, and 1 MPa; whereas when the undulant angle was 20°, the normal stress was 0.1 MPa. Furthermore, the shear stress–displacement curves were slip curves. In all other instances, these curves were peak curves. Notably, the peak curves were mainly divided into two types, the multi-peak and single-peak curves. The analysis of the shear failure characteristics of the structural plane revealed that the slip, multi-peak, and single-peak curves corresponded to the slip failure mode, pure shear failure, and shear failure accompanied by the compression-induced fracture, respectively.

The slip curve reflects that the slip failure of the structural plane is a cumulative damage process when the normal stress and undulant angle are small with unobservable fracture failure. During pure shear failure, the undulating body was sheared multiple times during the shearing process and the shear displacement was nonuniform, which caused a sudden increase in the shearing displacement, therefore, the shear stress–displacement curve with the multi-peak. When the normal stress and undulant angle were large, the undulating body produced cracks or was crushed in the initial stage owing to the stress concentration at the locking section and crushing zone. This caused the entire undulating body to be directly sheared and destroyed under the shear stress, thereby resulting in a shear stress–displacement curve with a single peak.

## Shear strength model of rock mass structural planes

### Peak shear strength

The Mohr–Coulomb equation is as follows:

2 $$\tau =c+{\sigma }_{n}\mathrm{tan}\left(\varphi \right),$$

where τ is the shear stress, ${\sigma }_{n}$ is the effective normal stress, c is the cohesion, ${\sigma }_{n}$ is the normal stress, and φ is the total friction angle. The peak shear strength envelopes for non-planar rock joints are some differences between the Coulomb relationship and the actual situation of rough structure plane shearing^69^. Furthermore, several previous studies have shown that the shear strength of a structural plane is related closely to normal stress and roughness^39,60,70^ and cohesion can be ignored when calculating the shear strength of structural surfaces^71^.

In addition, the shear resistance of the unfilled natural structural plane is mainly caused by the undulation of this plane and friction in the contacts. Therefore, the friction angle can be expressed as

3 $$\mathrm{\varphi }={\varphi }_{b}+\mathrm{\alpha },$$

where ${\varphi }_{b}$ is the basic frictional angle and $\mathrm{\alpha }$ is the undulant angle of the structural plane. Furthermore, for a flat structural plane, $\varphi ={\varphi }_{b}$*.*

**Figure 8:**
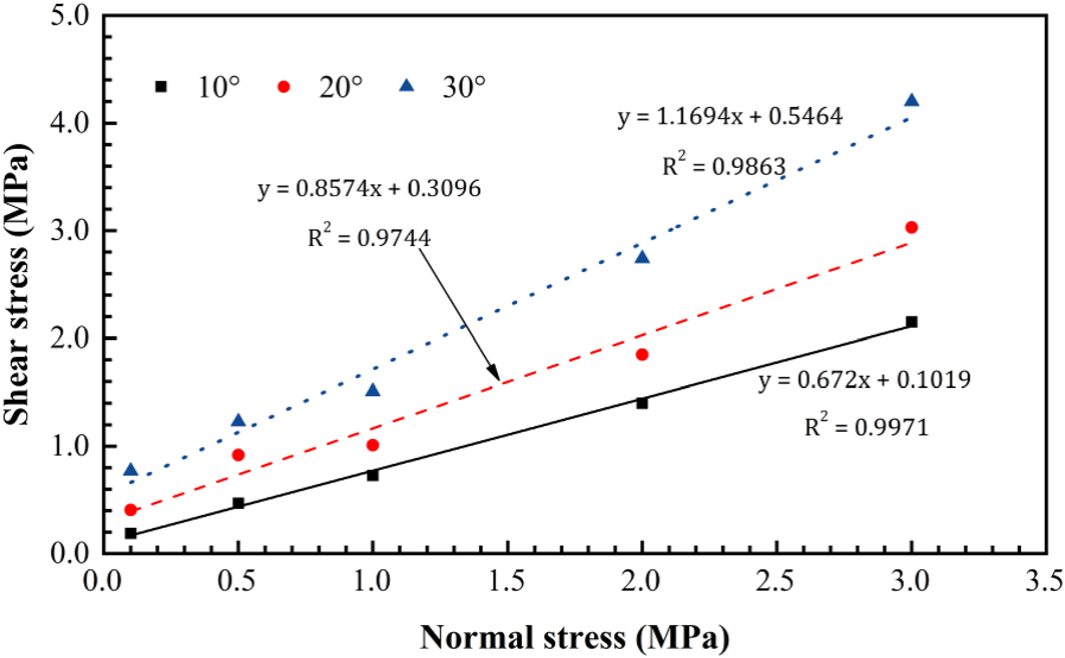
Peak shear strength trend as a function of the normal stress.

**Figure 9:**
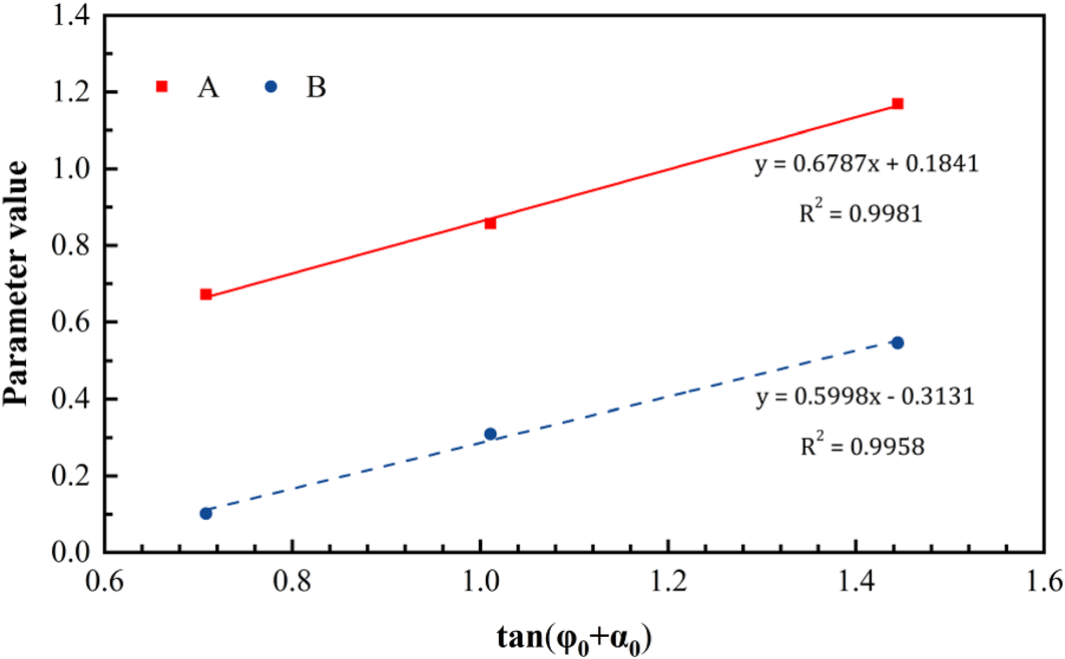
Trends of parameters A and B as a function of $\hbox{tan}\left({\varphi }_{0}+{\alpha }_{0}\right)$.

When the change in the basic friction angle during the shear process is disregarded, the initial undulant angle directly influences the friction angle for the same type of rock mass structural plane. Based on the direct shear test results obtained from the simulation that accounts for the normal stress and initial undulant angle, a good relationship between the normal stress, initial undulant angle, and peak strength is found to exist (Fig. 8) as follows:

4 $$\tau =A{\sigma }_{n}+B$$

where $\tau$ is the peak shear strength, ${\sigma }_{n}$ is the normal stress, and $A$ and $B$ are the coefficients related to the initial undulant angle. Additionally, coefficients $A$ and $B$ only consider fitting with $\mathrm{tan}({\varphi }_{0}+{\alpha }_{0})$. An optimal linear relationship is indicated between $A$, $B$, and $\mathrm{tan}({\varphi }_{0}+{\alpha }_{0})$ (Fig. 9):

5 $$A={C}_{1}\mathrm{tan}\left({\varphi }_{0}+{\alpha }_{0}\right)+{D}_{1}$$

6 $$B={C}_{2}\mathrm{tan}\left({\varphi }_{0}+{\alpha }_{0}\right)+{D}_{2},$$

where ${\varphi }_{0}$ is the initial basic friction angle; ${\alpha }_{0}$ is the initial undulant angle; and ${C}_{1}$, ${C}_{2}$,${D}_{1}$, and ${D}_{2}$ are the fitting coefficients.

Combining Eqs. (5) and (6), Eq. (4) can be expressed as follows:

7 $$\tau =\left[{C}_{1}\mathrm{tan}\left({\varphi }_{0}+{\alpha }_{0}\right)+{D}_{1}\right]{\sigma }_{n}+\left[{C}_{2}\mathrm{tan}\left({\varphi }_{0}+{\alpha }_{0}\right)+{D}_{2}\right]$$

According to the fitting results of the test data, the empirical equation of the shear peak strength of the structural plane is as follows:

8 $$\tau =\left[0.6787\mathrm{tan}\left({\varphi }_{0}+{\alpha }_{0}\right)+0.1841\right]{\sigma }_{n}+\left[0.59987\mathrm{tan}\left({\varphi }_{0}+{\alpha }_{0}\right)-0.3131\right]$$

Then, Eq. (8) was rewritten as follows:

9 $$\tau =\left[0.1841{\sigma }_{n}-0.3131\right]+\left[0.6787{\sigma }_{n}+0.59987\right]\mathrm{tan}\left({\varphi }_{0}+{\alpha }_{0}\right)$$

such that the following are obtained:

10 $${K}_{n\tau }=0.1841{\sigma }_{n}-0.3131$$

11 $${K}_{n}=0.6787{\sigma }_{n}+0.59987$$

Next, Eq. (9) was expressed as follows:

12 $$\tau ={K}_{n\tau }+{K}_{n}\mathrm{tan}\left({\varphi }_{0}+{\alpha }_{0}\right)$$

where ${K}_{n}$ denotes the correction coefficient of the friction coefficient corresponding to the structural plane, which is related to the normal stress, and ${K}_{n\tau }$ denotes the correction coefficient of the shear strength of the structural plane related to the normal stress.

### Post-peak shear strength

Grasselli and Egger^39^ and Lee et al.^38^ demonstrated that the shear stress curves from peak to residual shear values are close to a hyperbola. Furthermore, Grasselli and Egger^39^ proposed that an increase in the relative displacement results in a decrease in the friction coefficient as a hyperbolic function:

13 $$f={f}_{r}+\left({f}_{p}-{f}_{r}\right)\frac{{l}_{p}}{l}$$

14 $$f=tan\varphi $$

15 $${f}_{r}=tan{\varphi }_{r}$$

16 $${f}_{p}=tan{\varphi }_{p},$$

where ${l}_{p}$ is the shear displacement corresponding to the peak point of the shear stress, $l$ is the shear displacement, ${f}_{r}$ is the residual friction coefficient, ${f}_{p}$ is the peak friction coefficient, ${\varphi }_{r}$ is the residual friction angle, and ${\varphi }_{p}$ is the peak friction angle. Therefore, the following expression can be obtained:

17 $$tan\varphi =tan{\varphi }_{r}+\left(tan{\varphi }_{p}-tan{\varphi }_{r}\right)\frac{{l}_{p}}{l}$$

According to the elastic theory, the shear strength of the serrated rock mass structural plane mainly comprises the resistance caused by the friction angle of the rock. Based on the Eqs. (2) and (17) and ignoring the cohesion, the structural plane shear strength after peak shear can be expressed as follows:

18 $$\tau ={\tau }_{r}+\left({\tau }_{p}-{\tau }_{r}\right)\frac{{l}_{P}}{l},$$

where ${\tau }_{r}$ is the residual shear strength and ${\tau }_{p}$ is the peak shear strength.

**Figure 10:**
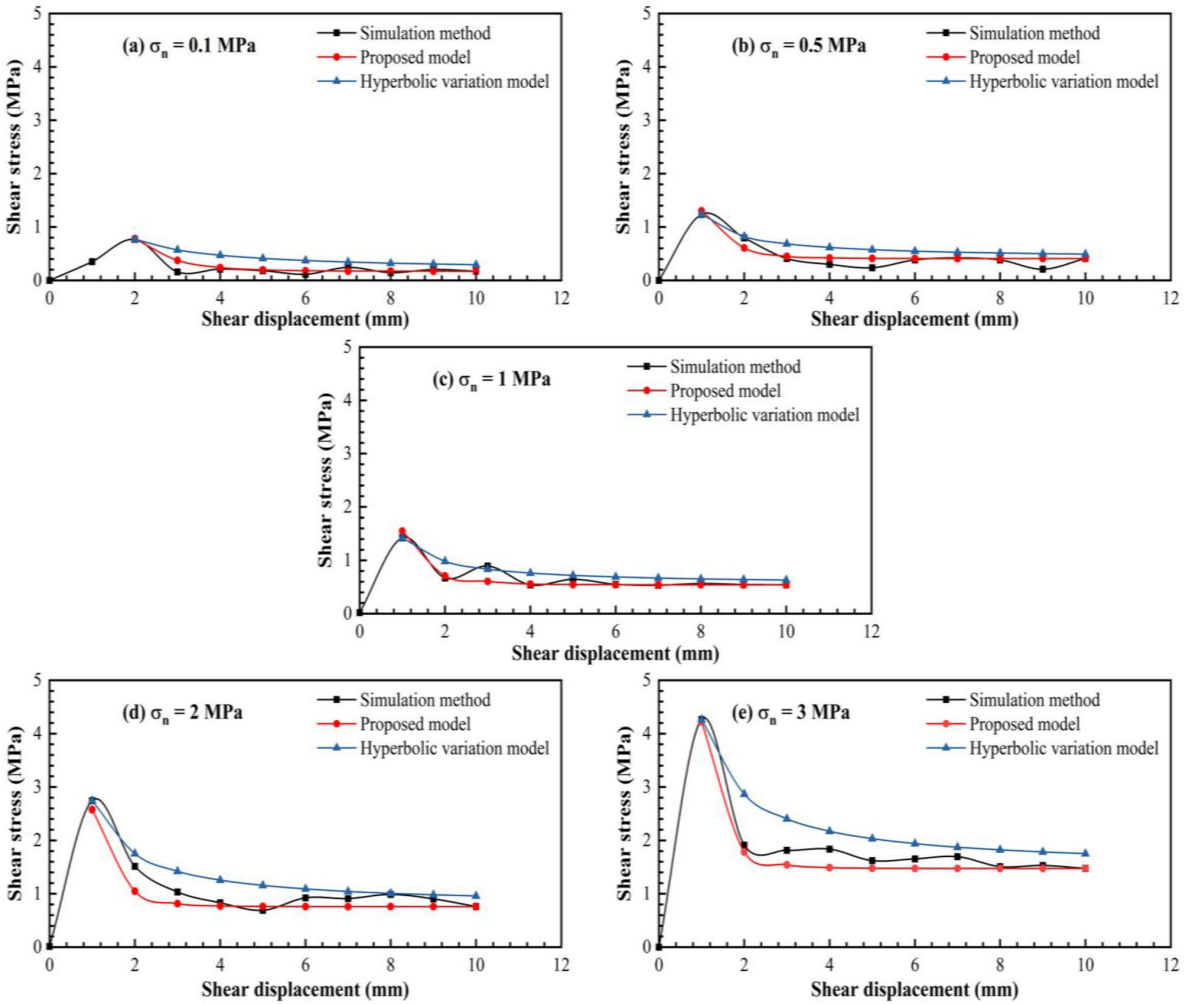
Comparison between the shear stress–displacement curves after the peak shear strength.

Considering the physical direct shear test of the structural plane with an undulant angle of 30° as an example, the variation trend in the shear stress under different normal stresses is obtained using the hyperbolic variation model. The calculation results obtained using the hyperbolic variation model proposed by Grasselli and Egger^39^ were found to be larger than and deviating significantly from the physical direct shear test results (Fig. 10).

Therefore, according to the experimental results and hyperbolic variation model proposed by Grasselli and Egger^39^, as well as repeated trial calculations, the equation for the post-peak shear strength can be improved as follows:

19 $$\uptau ={\uptau }_{\mathrm{r}}+{\mathrm{Ae}}^{\left[-(l-{l}_{p})/\mathrm{B}\right]},$$

where ${\tau }_{r}$ is the residual shear stress, ${l}_{p}$ is the shear displacement corresponding to the peak point of the shear stress, $l$ is the shear displacement, and $A$ and $B$ are the fitting coefficients.

According to Fig. 10, the fitting equations for the structural plane with an initial undulant angle of 30° under different normal stresses are as follows:

0.1 MPa:

20 $$\uptau ={\uptau }_{\mathrm{r}}+0.67{\mathrm{e}}^{\left[-(l - {l}_{p})/0.89\right]}$$

0.5 MPa:

21 $$\uptau ={\uptau }_{\mathrm{r}}+0.87{\mathrm{e}}^{\left[-(l - {l}_{p})/0.88\right]}$$

1 MPa:

22 $$\uptau ={\uptau }_{\mathrm{r}}+0.875{\mathrm{e}}^{\left[-(l - {l}_{p})/0.86\right]}$$

2 MPa:

23 $$\uptau ={\uptau }_{\mathrm{r}}+1.95{\mathrm{e}}^{\left[-(l - {l}_{p})/0.78\right]}$$

3 MPa:

24 $$\uptau ={\uptau }_{\mathrm{r}}+2.70{\mathrm{e}}^{\left[-(l - {l}_{p})/0.68\right]}$$

**Figure 11:**
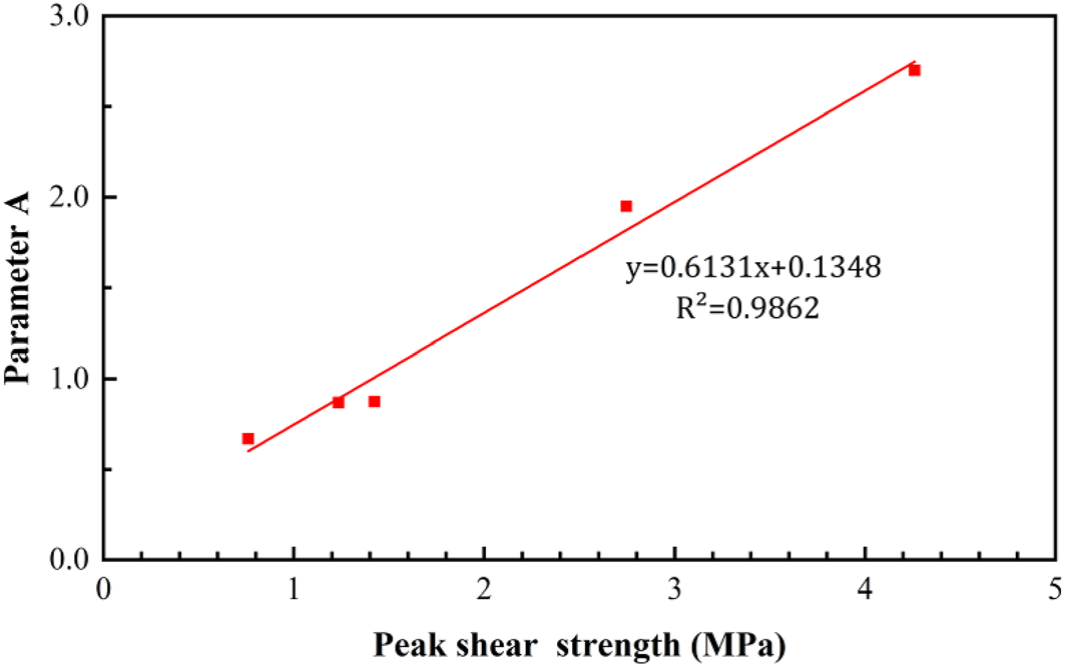
Trend of parameter A as a function of the peak shear strength.

**Figure 12:**
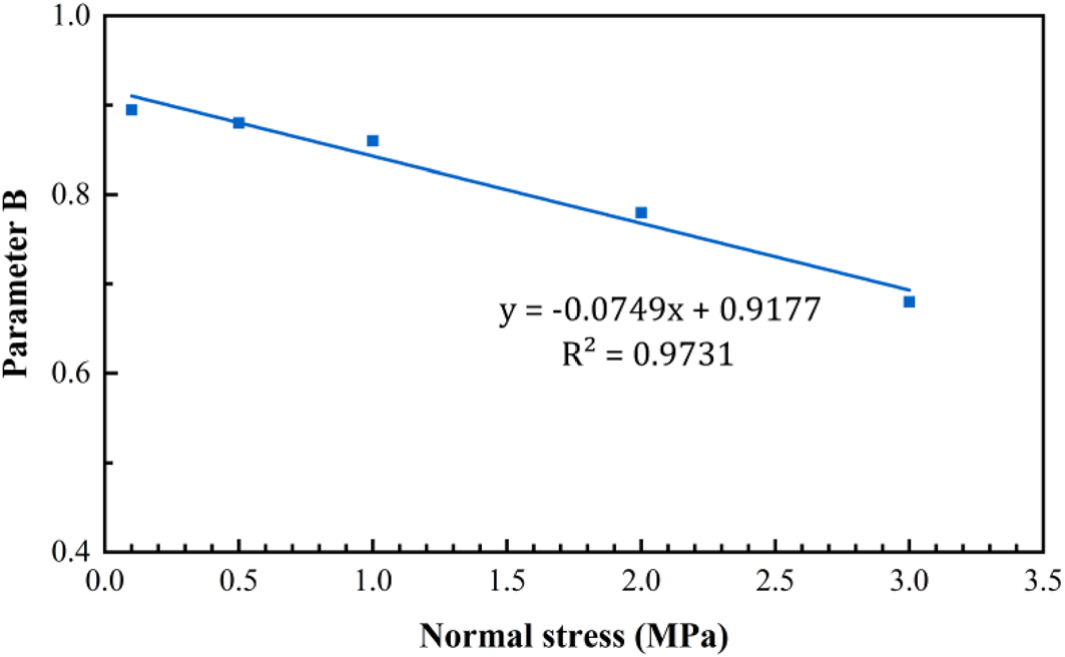
Trend of parameter B as a function of the normal stress.

According to Eqs. (20)–(24), coefficients A and B have a good linear relationship with the peak shear strength and normal stress, respectively (Figs. 11 and 12).

25 $$A=0.6131{\tau }_{p}+0.1348$$

26 $$B=-0.0749{\sigma }_{n}+0.9177$$

Based on Eqs. (19), (25), and (26), the post-peak shear strength can be expressed as follows:

27 $$\uptau ={\uptau }_{\mathrm{r}}+(0.6131{\tau }_{p}+0.1348){e}^{[-(l - {l}_{p})/(-0.0749{\sigma }_{n} + 0.9177)]}$$

## Shear strength verification

### Physical experiments

The data from the sandstone serrated structural planes studied by Cao^72^ were used in our analyses. A similar material with a mass ratio of high-strength gypsum: water: retarder = 1:0.25:0.005 was used to construct the structure plane samples. Table 4 lists the basic mechanical parameters of the similar material and sandstone. The mechanical parameters of the selected similar materials were equivalent to those of sandstone.

**Table 4:** Mechanical parameters of sandstone and similar materials^72^.

Material	Density (g cm^−3^)	Uniaxial compressive strength (MPa)	Cohesion (MPa)	Friction angle (°)	Elastic modulus (GPa)	Poisson's ratio
Sandstone	2.130	39.760	5.157	58.312	29.102	0.225
Similar materials	2.066	38.800	5.300	60.030	28.791	0.230

**Figure 13:**
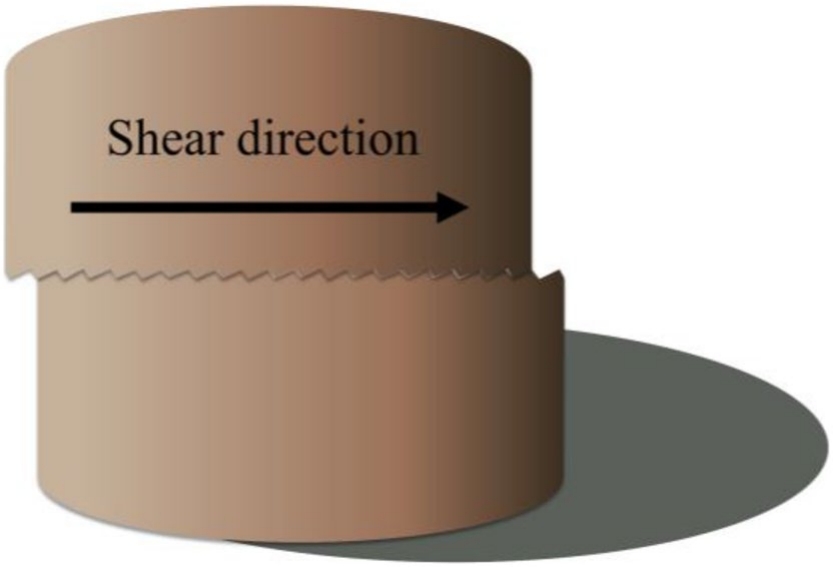
Schematic diagram of the test sample with a structural plane^72^.

**Figure 14:**
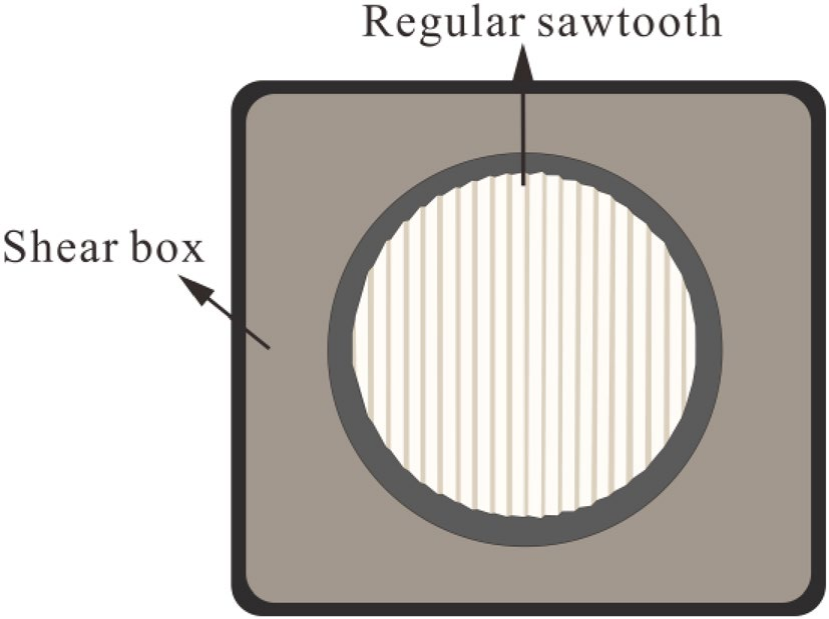
Plane diagram of the serrated structural plane in the test sample.

**Figure 15:**
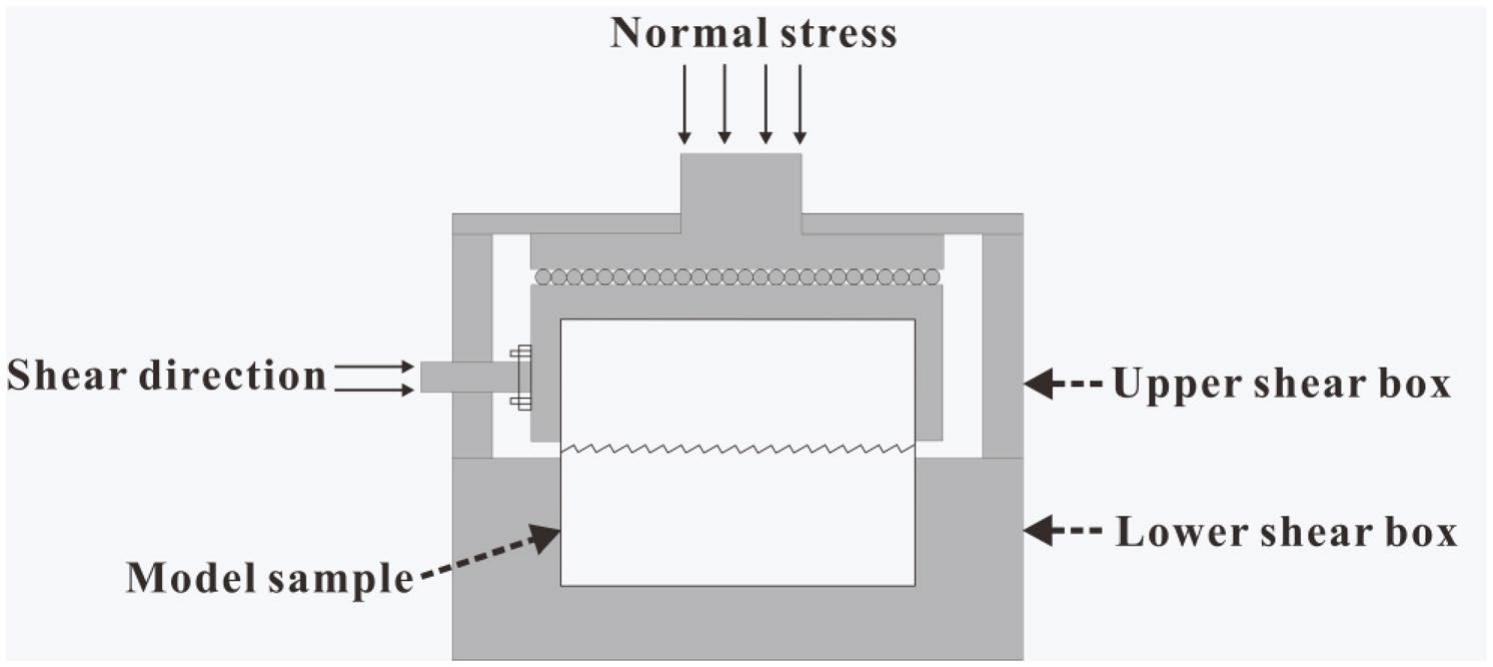
Shear box model.

The sample was cylindrical, and the structural plane with an undulant angle of 30° was located in the middle of the cylinder (Fig. 13). Figure 14 shows a diagram of the serrated structural plane of the test sample. The TJXW-600 microcomputer-controlled direct shear seepage coupling system developed by Changsha Yaxing Numerical Control Technology Co., Ltd. was used to conduct the rock mass structural plane shear test^72^. Figure 15 shows a schematic diagram of the shear box. The shear tests were performed using different normal stresses (1.27, 1.91, and 2.55 MPa) with a shear rate of 15 mm/min and shear displacement of 32 mm.

**Figure 16:**
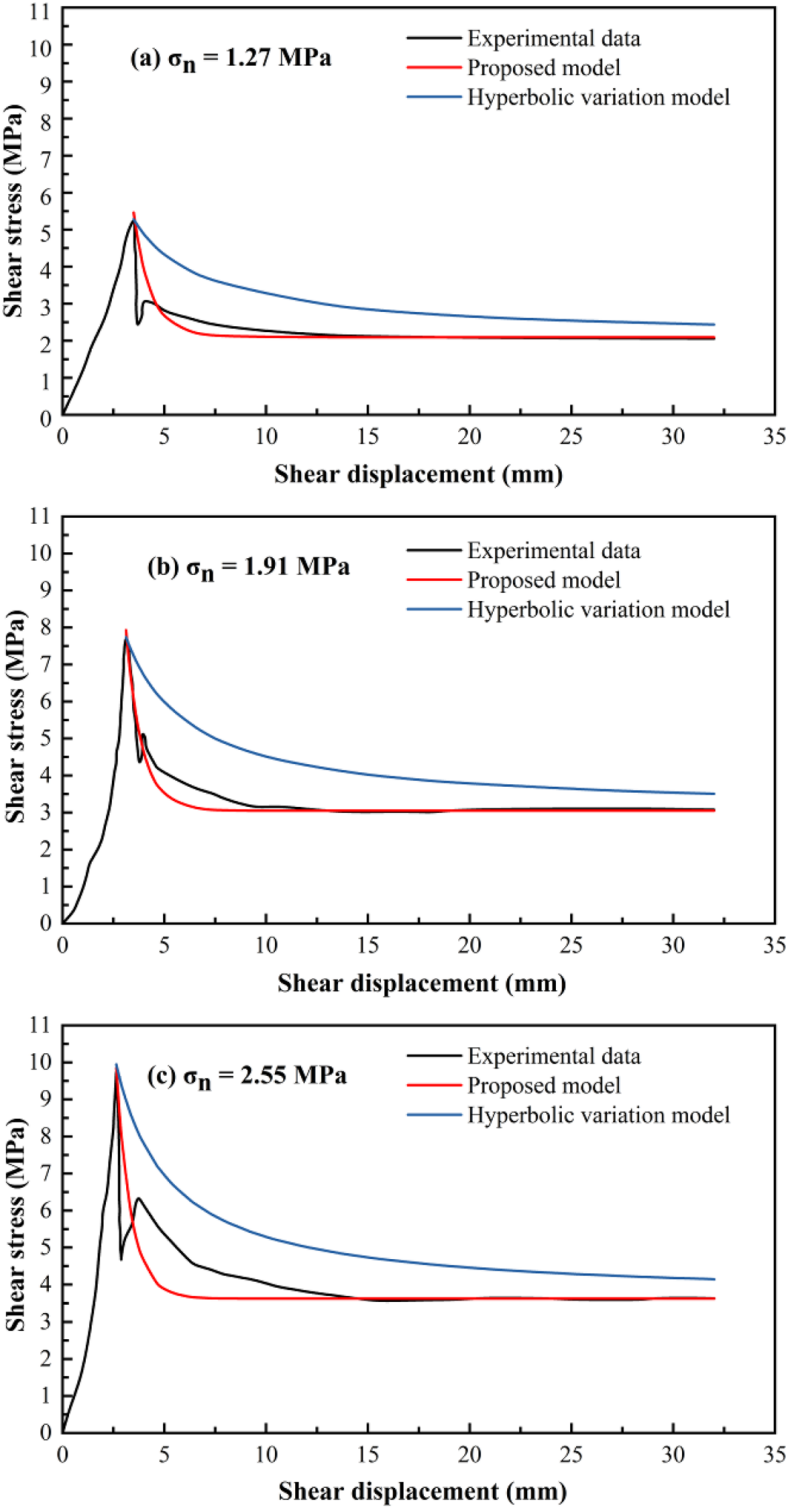
Comparison between the calculated post-peak shear stress values as a function of the shear displacement.

The shear strength–displacement curves are shown in Fig. 16. Additionally, the direct shear data from the artificial serrated structural plane obtained by Cao^72^ were used to verify the shear strength calculation model proposed in this study.

### Comparative analysis

#### Background

To verify the effectiveness of the shear strength model proposed in this study, the peak shear strength obtained experimentally was compared with the values calculated using the proposed model, as well as the models proposed by Parton^19^ and Shen^27^. The post-peak shear strength obtained experimentally was compared with the values calculated using the hyperbolic variation model proposed by Grasselli and Egger^39^ and that proposed in this study. The peak shear strength models proposed by Parton^19^ and Shen^27^ are briefly introduced below. The post-peak shear strength obtained using the hyperbolic variation model proposed by Grasselli and Egge^39^ is discussed in “Evolution characteristics of shear stress” section.

**(1) Patton model**

Newland and Allely^73^ first proposed the following equation to represent the shear strength of a structural plane:

28 $$\tau ={\sigma }_{n}tan\left({\varphi }_{b}+i\right)$$

Based on the equation proposed by Newland and Allely^73^, Partton^19^ and Goldstein et al.^74^ proposed the following equation for the peak shear strength of a serrated structural plane as follows:

29 $$\tau ={\sigma }_{n}\mathrm{tan}({\varphi }_{b}+\mathrm{\alpha })$$

In Eqs. (28) and (29), ${\sigma }_{n}$ is the normal stress, ${\varphi }_{b}$ is the friction angle corresponding to the smooth structural plane, *i* is the average deviation angle of particle displacements from the applied shear stress direction, and $\mathrm{\alpha }$ is the undulant angle of the structural plane.

**(2) Shen model**

Shen and Zhang^27^ proposed an empirical equation according to experimental tests and the Patton model:

30 $${\tau }_{p}={\sigma }_{n}\mathrm{tan}\left({\varphi }_{b}+{K}_{\beta }\beta \right)+{K}_{c}\alpha ,$$

where ${\tau }_{p}$ is the peak shear strength, ${\sigma }_{n}$ is the normal stress, ${\varphi }_{b}$ is the basic friction angle, $\alpha$ is the undulant angle of the regular structural plane, ${K}_{\beta }$ is the correction coefficient of the comprehensive internal friction angle of the structural plane, and ${K}_{c}$ is the correction coefficient for the comprehensive cohesion of the structural plane. When the normal stress is 0, ${K}_{c}$ is also 0. Based on the results of the structural shear test conducted by Shen and Zhang^27^, ${K}_{\beta }$ = 0.21 and ${K}_{c}$ = 0.038.

#### Comparison between the results

**(1) Peak shear strength**

Table 5 summarizes the peak shear strength values obtained using the physical direct shear test, Parton model, Shen model, and our proposed model. The errors between the peak shear strength calculated using the Patton model and that obtained from the physical direct shear test were discrete. Additionally, an increase in the normal stress increased the error, and the maximum error was 5.6%. This indicated that the Patton model may not apply to the shear of a structural plane under high normal stress. Furthermore, the results calculated using the Shen model differed substantial from those obtained using the physical direct shear test, the Patton model, and the proposed model. This result was ascribed to the correction coefficients proposed in the Shen model not being specifically applicable to structural planes with a certain lithology because they were obtained from a concrete structural plane sample^27^. The error between the peak shear strength calculated using the proposed model and that using the direct shear test was < 3%, thereby demonstrating the feasibility of our proposed model.

**Table 5:** Comparison between the shear stress calculation results.

Normal stress (MPa)	Test peak strength (MPa)	Initial basic friction angle (°)	Initial undulant angle (°)	Proposed model (MPa)	Parton model (MPa)	Shen model (MPa)	Error between test results and proposed model (%)	Error between test results and Parton model (%)
1.27	5.28	46.4	30	5.42	5.25	2.81	2.65	0.57
1.91	7.74	46.4	30	7.87	7.89	3.65	1.67	1.90
2.55	9.95	46.4	30	9.79	10.54	4.49	1.60	5.60

**(2) Verification of the post-peak shear strength model**

Figure 16 shows the variation trend of the post-peak shear stress with the shear displacement obtained using the physical direct shear test and that calculated using the proposed and hyperbolic variation models. The shear strength obtained using the hyperbolic variation model was higher than those obtained using the proposed model and physical direct shear test. Although the post-peak shear strength calculated using the hyperbolic model was too large, its variation trend was similar to those obtained from the experimental results. This was probably owing to the hyperbolic variation function proposed based on the direct shear test of natural structural planes, and the experimental results obtained using the natural structural planes have increased randomness and uncertainty; therefore, the accuracy of the calculation model obtained based on the experimental results of the natural structural planes needs to be improved.

The proposed model was improved based on the hyperbolic variation function. The variational trend of the post-peak shear strength obtained using the improved function was essentially consistent with the experimental results, and the calculated values were close to those obtained from the real experimental results. Moreover, the fitting degree of the results obtained using the proposed model was superior to that obtained using the hyperbolic variation model, thereby demonstrating the feasibility of the proposed calculation model for determining post-peak shear stress.

## Discussion

The verification of the peak shear strength revealed that the model proposed by Patton^19^ may not apply to the shear of a structural plane under high normal stress. In fact, by applying the statistical analysis of several shear test results of structural planes, Barton^75^ also reported that the Patton model yielded a large error under high normal stress; however, this model demonstrated high accuracy under low normal stress. Based on this, Table 5 reveals that the error between the experimental results and the results obtained using the proposed model increases with the decrease in the normal stress, and under low normal stress, such as 1.27 MPa, the error between the experimental results and the results obtained using the Parton model (0.57%) is much smaller than that between the experimental results and the results obtained using the proposed model (2.65%). This is because the structural plane was mainly subject to sliding failure under low normal stress, and the Patton model was mainly proposed for sliding failure. Based on the above analysis, this study suggested that the Patton model should be used to calculate the peak shear strength of the structural plane under low normal stress. In contrast, when calculating the peak shear strength of the structural plane under high normal stress, using the calculation method proposed in this study is a better alternative.

Notably, in this study, the proposed calculation models were only based on the test and verification of the structural planes of the sandstone rock mass. Accordingly, the coefficients of the equations proposed in this study might be appropriate only for calculating sandstone or mudstone rock masses. For other types of rock masses, particularly hard rock, such as granite, the values of the related parameters must be re-determined through experimentation. In future research, the shear strength of structural planes with different lithologies must be studied. The calculation model and particularly, the relevant parameters of the calculation model proposed in this study can be improved using statistical analysis of a large amount of experimental data.

In addition, this study ignored the influence of the second-order undulations on the shear strength of the structural plane. However, many previous studies have revealed that the classic rock mass shear strength model does not consider the influence of the second-order undulations and underestimates the shear strength when the normal stress is low^76–79^. Therefore, in future research, when studying the influence of the shear characteristics of the rock mass structural plane under low normal stress, the combined effects of the first and second-order undulations on the structural plane must be considered and a shear constitutive model must be established.

## Conclusion

In this study, the PFC2D numerical simulation method was used to conduct shear tests on the structural planes of rock masses with undulant angles of 10°, 20°, and 30°. The failure characteristics of the structural planes during the shear processes and the evolution characteristics of the shear stress with the change in the shear displacement were analysed in-depth. Based on the analysis of the shear test results, a peak shear strength model considering different undulant angles and normal stresses was proposed, and the hyperbolic function post-peak shear strength model was improved. The following conclusions were drawn:

1. During the shear process of the structural plane, with the increase in the undulant angle and normal stress, the shear failure of the structural plane was no longer a simple slip failure and shear failure but a shear failure accompanied by a compression-induced fracture phenomenon.
2. For the peak shear strength, although the calculation method of the Patton model is highly accurate under low normal stress, the calculation error of the low and high normal stress conditions of the proposed method was within an acceptable range. When calculating the peak shear strength of the structural planes under high normal stress, applying the proposed calculation method is a better option.
3. For the post-peak shear strength, the improved hyperbolic variational function had a better fitting degree. Additionally, the calculated values obtained using the improved method were close to those obtained experimentally.
4. In future research, the shear strength of structural planes with different lithologies must be studied. Additionally, the combined effects of the first and second-order undulations must be simultaneously considered on the structural planes and a shear constitutive model must be established.

## Data Availability

The data that support the findings of this study are available on request from the corresponding author.
